# The effect of dexamethasone on uterine receptivity, mediated by the ERK1/2-mTOR pathway, and the implantation window: An experimental study

**DOI:** 10.18502/ijrm.v20i1.10408

**Published:** 2022-02-18

**Authors:** Behrooz Niknafs, Naser Shokrzadeh, Mohammad Reza Alivand, Mohammad Bakhtiar Hesam Shariati

**Affiliations:** ^1^Immunology Research Center, Faculty of Medicine, Tabriz University of Medical Sciences, Tabriz, Iran.; ^2^Infertility and Reproductive Health Research Center, Health Research Institute, Babol University of Medical Sciences, Babol, Iran.; ^3^Department of Genetics, Faculty of Medicine, Tabriz University of Medical Sciences, Tabriz, Iran.; ^4^Department of Anatomical Sciences and Histology, School of Medicine, Kurdistan University of Medical Sciences, Sanandaj, Iran.

**Keywords:** Endometrial receptivity, Dexamethasone, HB-EGF, MSX-1, mTOR, Mouse.

## Abstract

**Background:**

The role of glucocorticoids in implantation has been demonstrated.

**Objective:**

This study aimed to evaluate the effect of dexamethasone on endometrial receptivity.

**Materials and Methods:**

In this experimental study, 40 BALB/c female mice aged eight wk old weighing approximately 25.0 ± 1.4 gr were used. The mice were divided into four groups (n = 10/each) of control, dexamethasone (100 μg/kg, intraperitoneal injection), mammalian target of rapamycin (mTOR) inhibitor (PP242) (30 mg/kg, intraperitoneal injection), and dexamethasone and PP242. The endometrial epithelium of the mouse was separated to measure messenger RNA expression of heart and neural crest derivatives-expressed protein 2 (*HAND2*), Msh homeobox 1 (*Msx-1*), heparin binding epidermal growth factor (*HB-EGF*), microRNA (miRNA) Let-7a, miRNA-145 and miRNA-451, using real-time polymerase chain reaction. Also, protein expression of mammalian mTOR and eukaryotic translation initiation factor 4E-binding protein1 (4E-BP1) was measured using western blot.

**Results:**

The results revealed that the expression of *Msx-1*, *HAND2*, *HB-EGF*, miRNA-451, and miRNA-Let-7a was significantly decreased in the endometrium in the dexamethasone group compared to the control, while the expression of miRNA-145 in the endometrium was up-regulated. Additionally, the administration of PP242, known as an inhibitor of mTOR, was associated with significantly reduced expression of *Msx-1*, *HAND2*, *HB-EGF*, miRNA-451, and miRNA-Let-7a, while PP242 induced messenger RNA expression of miRNA-145.

**Conclusion:**

It appears that dexamethasone can diminish uterine receptivity during the implantation period, at least to some extent, through the alteration of particular genes that impact endometrial receptivity. Furthermore, the mTOR pathway seemingly showed an essential role in endometrial receptivity.

## 1. Introduction

Lack of implantation is one of the primary reasons for infertility in females (1). Previous reports have shown that adhesion and growth factors, transcription factors, cytokines, steroid hormones, and lipid mediators take part in the process of endometrial receptivity (2, 3).

Similarly to heparin binding epidermal growth factor (*HB-EGF*), it has been shown that the Msh homeobox 1 (*Msx-1*) gene is indispensable for implantation of the embryo, in encoding a member of the muscle segment homeobox gene family (4). Recently, it was found that the *Msx-1* gene has an essential role in implantation via alteration of the cellular microarchitecture in the uterus (5).

Heart- and neural crest derivatives-expressed protein 2 (*HAND2*) is a transcription agent found in the uterine stroma (6). It has also been shown that stromal *HAND2* intervenes in implantation by reducing the differentiation of uterine epithelial cells (7). microRNAs and their target genes also play an essential role in embryo implantation and growth (8). One of the irregular microRNAs in recurrent implantation failure is microRNA 145 (MiRNA-145) (9). MiRNA-451 has various physiological functions such as cell differentiation, cell proliferation, and migration/invasion into the cell. All of these processes are essential for the growth and survival of endometriosis implants (10). miRNA Let - 7a could influence the ability of implantation in stimulated blastocysts, thereby changing the expression of the gene (11).

Several signaling proteins including extracellular signal-regulated kinases (ERKs), protein tyrosine kinase 2 (PTK2), and mammalian target of rapamycin (mTOR) participate in the regulation and combination of these molecular and cellular procedures to ensure effective implantation (12). mTOR belongs to the phosphoinositide 3-kinases (PI3Ks)-related kinase superfamily; it also plays a major role during implantation (13). mTOR inhibitor (PP242) is capable of inhibiting both mTOR complex 1 (C1) and mTORC2 (14). This inhibitor blunts the activity of the eukaryotic translation initiation factor 4E - binding protein1 (4E-BP1) as well (15). Dexamethasone is an artificial steroid compound, possessing a high attraction to the glucocorticoid receptor (16). However, its effect(s) on the signaling pathways thought to be involved in implantation and uterine receptivity is not yet understood.

Therefore, in this study, we pursued several goals. Our first goal was to evaluate the effects of dexamethasone on endometrial receptivity by investigating molecular changes (in *HB-EGF*, *HAND2*, *Msx-1*, miRNA-145, miRNA-451 and miR-Let-7a). The second purpose of this study was to investigate the expression of the above genes under the influence of dexamethasone in the mTOR-4EBP1 signaling pathway. Finally, by controlling the mTOR-4EBP1 pathway using an mTOR inhibitor, we examined the effects of dexamethasone on uterine receptivity, which could reduce the effects of dexamethasone on uterine acceptance.

## 2. Materials and Methods

### Chemicals

The dexamethasone used for the in vitro testing was obtained from Sigma - Aldrich (St. Louis, MO, USA) and the PP242 was obtained from Selleckchem (Cat No. S2218; Houston, TX, USA). The Thermo Fisher Scientific kit (Cat No. EP0441; Waltham, MA, USA) was used for complementary DNA (cDNA) synthesis, and Amplicon Master SYBR Green (Cat No. 45323402; Odense, Denmark) was utilized for the real-time polymerase chain reaction (rt-PCR). TRIzol LS Reagent (Invitrogen; Carlsbad, CA, USA) was prepared for extraction of RNA and proteins. mTOR, p - mTOR, 4EBP1, p - 4EBP1 and β - actin antibody were obtained from Santa Cruz (Dallas, TX, USA).

### Animals

In this experimental study, 40 BALB/c female mice aged eight wk were obtained from the Razi Institute (Tehran, Iran). The mice were kept at a temperature of 21 ± 1°C, humidity of 50 ± 10%, and a 12-hr light/dark period with completely free access to food and water.

### Induction of pregnancy

Before mating, all mice were in the estrous cycle (17). Female mice (in the estrus stage) were matched with adult males in a separate cage during the night to mate. Confirmation of pregnancy was performed by seeing a vaginal plug in the female mice the next morning; this day was considered the first day of pregnancy.

### Study design 

After complete assurance of pregnancy, the mice were randomly divided into four groups (n = 10/each): 1) the control group (normal saline, dimethyl sulfoxide [DMSO], polyethylene glycol [PEG], TWEEN through intraperitoneal injection); 2) DEX: the dexamethasone group (100 μg/kg of dexamethasone); 3) PP242: the mTor inhibitor group (30 mg/kg of PP242); and 4) DEV+PP242: the mTor inhibitor and dexamethasone group (100 μg/kg of dexamethasone, 30 mg/kg of PP242). After isolating the female mice from the males, the gestational day was recorded. On day four and five of gestation, in the early morning after 8:00 A.M, the control group received the vehicle described above, whereas dexamethasone (100 μg/kg) (18) combined with a saline solution was injected intraperitoneally into the DEX group. PP242 (30 mg/kg) (19) was intraperitoneally injected at 8:00 A.M. into the mice in the mTOR inhibitor group (PP242) on the 4 ${}^{\mathrm{th}}$ and 5 ${}^{\mathrm{th}}$ day of gestation, and in the DEX+PP242 group, dexamethasone and PP242 were injected intraperitoneally. On day five of gestation, approximately two hr after the last injection, the mice were killed by cervical displacement under anesthesia. Uterine horn specimens were isolated under sterile conditions in the mice, and endometrial tissue which included epithelium and stroma cells were mechanically shaved and poured in TRIzol LS Reagent (Invitrogen; Carlsbad, CA, USA) and then were placed at -80°C to perform molecular tests.

### RNA isolation and quantitative RT-PCR

Total RNA was isolated from each sample by TRIzol reagent and the reverse transcription reaction was performed with an RT kit (Thermo Fisher Scientific) based on the instructions of the manufacturer. The cDNA was proliferated by real-time quantitative PCR with the SYBR Green Master Mix (Ampliqon; Odense, Denmark). The sequence of primers is shown in table I. To analyze samples, the threshold was designated according to the exponential stage of production, and to analyze the data, the 2 - ΔΔCt procedure was performed as explained previously (16). MRNA expression levels were normalized with glyceraldehyde 3-phosphate dehydrogenase mRNA. All reactions were performed in three replications.

### Micro RNA: Reverse transcription and RT-PCR

First, the whole RNA was separated from each sample, and then the reverse transcriptase reactions were performed using the Thermo Fisher Scientific enzyme. Overall, 2000 nanograms of the total RNA was reverse transcribed by the reverse thermocrypt transmitter Thermo Fisher kit. Using the manufacturer's instructions, the RT-PCR was done with the SYBR Green PCR Master Mix (Amplicon; Odense, Denmark) with a miRNA-special forward primer and a universal reverse primer, which are displayed in table II. Instructions for this reaction included 95°C for 5 min and the continuation of this reaction by 40 cycles of amplification at 95°C for 30 sec, 57°C for 30 sec, and 72°C for 30 sec. U6 (RNU6-1) small nuclear RNA was applied as an example of internal control, and eventually the 2 - ΔΔCt procedure was used to analyze the data.

### Western blot analysis

TRIzol LS was used to isolate total protein from endometrial tissue. Separated proteins were homogenized with 200 μl lysis radioimmunoprecipitation assay buffer comprising a protease inhibitor cocktail (Sigma - Aldrich; St. Louis, MO, USA), and after homogenizing, we centrifuged them at 12,000 g at 4°C for 20 min. The protein concentration was calculated using the Bradford method in the obtained sequence (Bio-Rad; San Francisco, CA, USA). An equivalent amount of total protein (100 μg) of each sample was separated using sodium dodecyl sulfate-polyacrylamide gel (SDS-PAGE; 10%) and all of these proteins were transferred to a polyvinylidene difluoride membrane. Non-proprietary connections were closed with 5% non-fat milk for two hr at laboratory room temperature. Then, the membranes were heaped with monoclonal primary antibodies (1:500; Santa Cruz): ERK1/2 (H - 72, sc - 292838), p - ERK1/2 (Thr 177, sc - 16981 - R), mTOR (sc - 1550 - R), p - mTOR (Ser 2481; sc - 293089), 4E - BP1 (sc - 997), p - 4E - BP1 (sc - 293124), and β - actin (sc - 47778) as a loading control (1:1000; Sigma Aldrich) overnight at 4°C. After washing with phosphate-buffered saline, the membrane was incubated with horseradish peroxidase-conjugated secondary anti-rabbit antibodies (1:5000; Santa Cruz) for one hr. Bands became visible by a boosted chemiluminescence diagnostic kit (Bio-Rad). The density and thickness of the desired bands were measured by the ImageJ software (https://imagej.nih.gov/ij/).

**Table 1:** mRNA primer sequences (5'-3') used in quantitative real-time PCR


**Primer name**		**Primer sequence (5 → 3)**
*Msx-1*		
	**Forward**	TCTCTTAAACCCCTTGCTACACAC
	**Reverse**	GGCCTCTGCACCCTTAGTTT
* HB-EGF*		
	**Forward**	CCTCTTGCAAATGCCTCCCT
	**Reverse**	CCTCCTCTCCTGTGGTACCTAAA
* HAND2*		
	**Forward**	CGACGTGAAAGAGGAGAAGAGG
	**Reverse**	CTGCTCTCCTCTTCTTCACTGC
*GAPDH*		
	**Forward**	AATGTGTCCGTCGTGGATCTGA
	**Reverse**	GATGCCTGCTTCACCACCTTCT
*Msx-1*: Msh homeobox 1, *HB-EGF*: Heparin-binding EGF-like growth factor, *HAND2*: Heart and neural crest derivatives expressed 2, *GAPDH*: Glyceraldehyde-3-phosphate dehydrogenase		

**Table 2:** miRNA primer sequences (5'-3') used in quantitative real-time PCR


**Primer name**		**Primer sequence (5 → 3)**
** miR-Let - 7a**		
	**STL**	GTCGTATCCAGTGCAGGGTCCGAGGTATTCGCACTGGATACGACACATCGT
	**Forward **	GGGTAACACTGTCTGGTAACGAT
**miR-145**		
	**STL**	GTCGTATCCAGTGCAGGGTCCGAGGTATTCGCACTGGATACGACAGGGAT
	**Forward**	TTGAACCCTCATCCT GTGAGCC
**miR- 451**		
	**STL**	CTCAACTGGTGTCGTGGAGTCGGCAATTCAGTTGAGAAA-CTCAG
	**Forward**	GGAAGATCTTGACAAGGAGGACAGGAGAG
**Universal**		
	**Reverse**	GTGCAGGGTCCGAGGT
**U6**		
	**Forward**	GCTTCGGCAGCACATATACTAAAAT
	**Reverse**	CGCTTCACGAATTTGCGTGTCAT
	**STL**	GTCGTATCCAGTGCAGGGTCCGAGGTATTCGCACTGGATACGACAAAAATAT
STL: Stem-loop, Let - 7a: Lethal-7, miR: MicroRNA, U6: RNU6-1		

### Ethical considerations

All steps were approved by the Ethics Committee of Tabriz University of Medical Sciences, Tabriz, Iran (Code: pirc.tbzmed.ac.ir; Grant number: 58538).

### Statistical analysis

The Statistical Package for the Social Sciences (SPSS), version 24 (IBM, International Business Machines Corp.; Armonk, NY, USA) was used to analyze the data and differentiate between the experimental groups. To analyze statistical importance, a two-way analysis of variance with Tukeyʼs post-hoc test was used. All data were expressed as mean ± standard error of the mean (SEM), and statistical significance was defined at p < 0.05.

## 3. Results 

### Results of real-time PCR 

**Figure 1:**
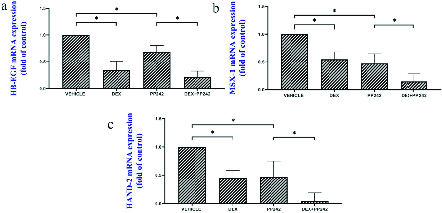
The relative mRNA expression of *HB-EGF* (a), *Msx-1* (b), *HAND2* (c) in the endometrium of different groups. Data are shown as Mean ± SEM (n = 3). *P < 0.05.

**Figure 2:**
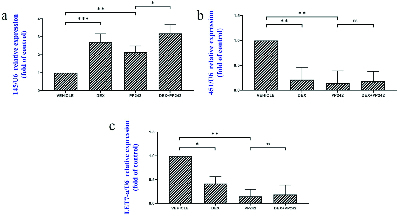
The relative microRNA expression of miR-145 (a), miR-451 (b), Let-7a (c) in the endometrium of different groups. Data are shown as Mean ± SEM (n = 3). *P < 0.05, **P < 0.01, ***P < 0.001.

The results of the real-time PCR revealed the influence of dexamethasone on the expression of *HB-EGF*. Our results showed that dexamethasone was associated with a markedly lower expression of *HB-EGF* compared to the control group (p = 0.03; Figure 1a). It was also demonstrated that the mRNA expression of the *Msx-1* gene was significantly lower in the DEX group compared to the control (p = 0.04; Figure 1b). *HAND2* mRNA expression after injection of dexamethasone was significantly different compared to the control group (p = 0.04), as is displayed in figure 1c. In addition, in the DEX group, miRNA-145 expression was considerably higher in comparison with the control group (p ≤ 0.001; Figure 2a). However, in the DEX group, miRNA-451 and miRNA Let-7a expression were considerably lower in comparison with the control group (p ≤ 0.001, Figure 2b and p = 0.04, Figure 2c, respectively).

The outcomes of the gene expression analysis showed the influence of PP242 on the expression of the *HB-EGF* gene. According to the obtained data, PP242 significantly changed the expression of the *HB-EGF* gene in the PP242 group compared to the control (p = 0.04) (Figure 1a). Our findings revealed that the mRNA expression of *Msx-1 *was significantly altered in the PP242 group in comparison with the control group (p = 0.04, Figure 1b). As revealed in figure 1c, *HAND2* mRNA expression after PP242 injection was considerably different compared to the control group (p = 0.04). However, in the PP242 group, miRNA-145 expression was considerably higher in comparison with the control group (p ≤ 0.001; Figure 2a). In addition, in the PP242 group, miRNA-451 and miRNA-Let-7a expression were both considerably lower compared to the control group (p ≤ 0.001, figure 2b and 2c, respectively).

Moreover, the expression of *HB-EGF* (p = 0.03, Figure 1a), *Msx-1* (p = 0.02, Figure 1b), and *HAND2* (p = 0.01, Figure 1c) were significantly different when the DEX+PP242 and PP242 groups were compared. In the DEX+PP242 group, miRNA-451 (Figure 2b) and miRNA-Let-7a (Figure 2c) expression were similar to that of the PP242 group, but the expression of miRNA-145 was considerably lower in comparison with the group which only received PP242 (p = 0.04, Figure 2a).

### Western blot 

**Figure 3:**
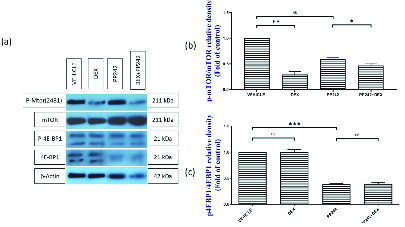
Total protein expression and phosphorylation level of mTOR and 4E-BP1 in the uterus. (a) Representative images of mTOR, 4E-BP1 and β-Actin proteins. The relative phosphorylation levels of mTOR (b) and 4E-BP1 (c) in the endometrium. Data are presented as Mean ± SEM (n = 3). *P < 0.05, **P < 0.01, ***P < 0.001.

The western blot analysis (Figure 3a, b) demonstrated that the administration of dexamethasone in the DEX-treated group was associated with significantly lower mTOR phosphorylation compared to the control (p ≤ 0.001). However, the difference in the level of phosphorylated 4E-BP1 protein between the DEX group and control group was not statistically significant (p = 1.00; Figure 3a, c). The western blot analysis also showed that the administration of PP242 did not significantly decrease the rate of mTOR phosphorylation in the PP242 group compared with the control (p = 0.68). Further analysis also revealed that in the DEX+PP242 group, the level of p-mTOR was lower in comparison with the PP242 group (p = 0.08; Figure 3a, b). Moreover, the administration of PP242 was associated with considerably lower levels of p-4E-BP1 in the endometrium of the PP242 group compared with the control group (p ≤ 0.001, Figure 3a, c). Notably, the level of p-4E-BP1 was not significantly different between the DEX+PP242 and PP242 groups (p = 1.00; Figure 3a, c).

## 4. Discussion

In our previous study, evaluation of endometrial morphology indicated that the administration of dexamethasone could lead to a loss of differentiation in the epithelial cells in the uterus, a decrease in the proliferation of stromal cells, and could prevent decidualization after blastocyst adhesion (16). Inhibitors of the mTOR protein can decrease the unceasing production of endometrial epithelial cells and elevate glycoprotein levels on epithelial cells, resulting in unsuccessful receptivity. In the present study, the results demonstrated that dexamethasone lowered the expression of *HB-EGF*, *Msx-1*, and miR-Let-7a in the endometrium of mice.

*HB-EGF* is a member of the EGF family, and plays a vital role in the `molecular crosstalk' between the embryo and intima. It has been found that *HB-EGF* is regularly expressed in endometrial tissue, particularly in the glandular and luminal epithelium, reaching its zenith of expression at day 20 or the mid-secretory stage, menstrual cycle, decidua, and placenta in the whole menstrual cycle (20). This is the first study performed on the impact of dexamethasone on *HB-EGF* expression. The *HB-EGF* gene expression is increased in the rat uterine epithelium about six to seven hr before the implantation process (21).

We found that after the administration of PP242, the *HB - EGF* gene was not significantly downregulated. These findings indicated that the mTOR signal pathway presumably had an important effect on the downregulation of the *HB-EGF* gene after the treatment of mice with dexamethasone. Additionally, the co-administration of dexamethasone and PP242 in pregnant mice resulted in a slight decrease in the expression of *HB-EGF*. Once the mTOR signaling pathway was suppressed using PP242, the phosphorylation of 4EBP1 did not occur in the experimental groups. The downregulation of *HB-EGF* was more pronounced after the co-administration of dexamethasone and PP242, reflecting the inhibitory effects of dexamethasone on the expression of *HB - EGF*. Hence, it would be plausible that the reduced expression of *HB-EGF* precipitated by dexamethasone is through the mTOR. The administration of dexamethasone on the 4 ${}^{\mathrm{th}}$ day of pregnancy was also able to slightly change the expression of *Msx-1* during the implantation window. The simultaneous administration of dexamethasone and PP242 resulted in minimal alteration of the *Msx-1* gene during the implantation window.

According to the published literature, no study has been performed to evaluate the impact of dexamethasone on the expression of the *Msx-1* gene. In the gestation period, *Msx-1* is continually expressed in the glandular and luminal epithelium, mainly on the 4 ${}^{\mathrm{th}}$ day of gestation, which is the time of blastocyst adhesion (4, 22). A lack of *Msx-1* in the murine uterus can lead to infertility due to impairment in the implantation window. Luminal epithelial cells lacking the *Msx-1* gene are unable to pass through the extremely polarized columnar shape structure. Transitioning to a less polar cuboidal structure will facilitate the adhesion of blastocysts (23). The absence of the *Msx-1* gene leads to the production of fibroblast growth factor.

*HAND2*, a transcription factor that is stimulated by the hormone progesterone in the uterine stroma, has also been reported to be involved in receptivity, implantation, and decidualization in mice (24). In one study, it was shown that on day three of pregnancy, the expression of *HAND2* occurs strongly in the stroma, but not particularly in the epithelium. Stroma expression continues during implantation and can be detected through 8.5 days of gestation, indicating the role of *HAND2* during decidualization and implantation (24). Other studies have reported that removing *HAND2* from the uterus leads to infertility due to implantation defects (6). According to our study, prescribing dexamethasone and PP242 reduced *HAND2* gene expression in the mouse uterus in the implantation stage. However, there was an additive effect in the concomitant use of dexamethasone and PP242.

Mucins are located on the apical surface of the uterine epithelium, which provide a mechanical barrier against microbial attacks. Due to the anti-adhesion property of Mucin 1, decreased expression of *MUC-1* in the uterus will increase uterine implantation (25). The administration of dexamethasone before the uterine implantation window minimally changed the expression of miR-Let-7a in the pregnant mice. Considering the reduction in the expression of miR-Let-7a in response to the treatment with dexamethasone and/or PP242, it could be implied that a slight decrease of the miR-Let-7a, in response to the administration of dexamethasone, is mediated by the mTOR pathway. Numerous studies have been performed that show the importance of microRNAs in the regulation of embryo implantation. For instance, the significance of the miR-Let-7 family during the pre-implantation period in the uterus has been demonstrated (25).

It has been shown that miR-Let-7a can influence embryo implantation, at least to some extent, through the modulation of the expression of *MUC-1* in humans and mice. It has also been found that miR-Let - 7a is able to control implantation via targeting the mucin1 protein which is a major cell surface-associated glycoprotein, expressed in the uterus of mice (26).

One of the main genes targeted by miR-Let-7a in the uterus is *MUC - 1*; it directly binds to the untranslated region of the *MUC-1* gene, leading to increased expression of this gene in the first five days of gestation. Expression of the *MUC - 1* gene is locally reduced in the uterus, resulting in improved implantation (26).

The primary functions of miRNAs could be listed as: 1) binding to the 3 '- UTRs of mRNAs; 2) inducing the degradation of proteins; and 3) preventing the translation process. It is estimated that these types of molecules target 30-60% of all mammalian genes (27). It is suggested that dexamethasone is capable of reducing uterus receptivity and leading to an increase in the expression of *MUC-1*. Many studies have shown the beneficial effects of artificial glucocorticoids as a result of pregnancy (28). However, our results showed that dexamethasone decreased endometrial acceptance by regulating Let-7a miRNA so that Let-7a would target *MUC-1*.

Rong Li and colleagues showed through an extensive genome analysis a significant reduction in miRNA-451 expression in females who had in vitro fertilization with decreased endometrial receptivity because of rising concentrations of progesterone on the day of human chorionic gonadotrophin, compared to females with normal progesterone levels (29). Oestradiol-dependent up-regulation of miR-451 was also seen in mouse uteri (30). Another study also showed that miR-451 was up-regulated at the time of the implantation window, and by targeting mRNA Ankrd46, it plays an important role in embryo implantation (31). Thus, according to past results, it can be concluded that dexamethasone down-regulates miRNA-451 and leads to a reduction in implantation.

Regarding studies of microRNAs, Revel and colleagues showed that miR-145 had high levels of expression, while their anticipated targets, a group of adhesive molecules involved in embryo implantation, had low expression in a group of patients with defects in implantation and uterine acceptance. In addition, this study showed a threefold increase in the rate of miR-145 expression associated with recurrent implantation failure in patients with normal fertility (32). Other studies have also shown that miR-145 affects the attachment and adhesion of the embryo (10) by reducing the amount of IGF1 receptors in the endometrium (9). Therefore, according to this information, it can be concluded that dexamethasone can reduce uterine acceptability by increasing the expression of miR-145.

Several signaling pathways are thought to be involved in the expression of various genes and proteins during implantation. In the current study, we observed the impact of dexamethasone and PP242 on endometrial receptivity and that the mTOR/4E-BP1 pathway may have been involved in this scenario. In our study, PP242 was employed for investigating the mechanism by which dexamethasone can influence uterine receptivity through an alteration in the expression of *Msx-1*, *HB - EGF*, *MUC-1* and miR-Let - 7a, independent of the activation of the mTOR pathway. The impact of PP242 on the suppression of the mTOR signaling pathway and reduced expression of 4EBP1 (as a downstream protein) was confirmed by the inhibition of the mTOR signaling pathway in the mice, accompanied by a slight decrease in *Msx-1*, *HB - EGF*, *MUC-1* and miR-Let-7a.

These signaling pathways are capable of controlling the mobility and stimulation of trophoblast cell proliferation during fertilization which are vital for embryo implantation (33). Wang and colleagues identified a PKC - mediated pathway by which mTORC1 was activated via ERK1/2, which led to the stimulation of protein production. Hindrance of the mTOR pathway by using rapamycin can repress the production of proteins, mediated by the E2 protein, because of its dependence on the mTOR pathway in epithelial cells. The suppression of the mTOR pathway results in the emergence of remarkable physiological outcomes as this inhibits E2 - induced DNA production in murine and human endometrial epithelial cells (34). The results of the current study showed that dexamethasone is sufficient for changing endometrial receptivity, resulting from modulation of the mTOR pathway.

## 5. Conclusion

Our research has shown that dexamethasone reduces the expression of genes that impact mice uterine receptivity during implantation. It seems that such a decline in the expression of the above genes might be interceded by the mTOR signaling pathway, which could result in altered endometrial receptivity in normal cycles.

##  Conflict of Interest

The authors declare that there is no conflict of interest that prejudices the impartiality of this scientific work.
